# An investigation of the population impact of variation in HbA_1c _levels in older people in England and Wales: From a population based multi-centre longitudinal study

**DOI:** 10.1186/1471-2458-8-54

**Published:** 2008-02-11

**Authors:** Lu Gao, Fiona E Matthews, Lincoln A Sargeant, Carol Brayne, MRC CFAS

**Affiliations:** 1 MRC Biostatistics Unit, Institute of Public Health, Cambridge, UK; 2 Department of Public Health and Primary Care, Institute of Public Health, Cambridge University, Cambridge, UK; 3 MRC Cognitive Function and Ageing Study (corporate author)

## Abstract

**Background:**

Diabetes is common in the older population and is increasing. Glycated hemoglobin (HbA_1c_) is an indicator of average blood glucose concentration over the past three months. The HbA_1c _test is currently one of clinical methods used to check diabetes control. Recent studies have suggested diabetes is a risk factor for dementia, cognitive dysfunction and physical disability. In addition, there have reported the relationship between HbA_1c _and mortality on all cause, cardiovascular disease and cognitive function, but few studies have investigated the relationship concentrating on the older population.

The aim of this study is to investigate the association between the level of HbA_1c _and mortality from all causes, incident cardiovascular disease, cognitive decline and physical disability in people aged 65 and over in England and Wales.

**Methods:**

1139 men and women aged 69 years and over who were participants in a ten year population based ageing multi-centre, longitudinal study who had HbA_1c _measurements after 5–6 years of follow up. All participants were flagged for death notification including causes at the Office of National Statistics. Information on health including vascular conditions, cognitive status, physical function and dementia were available from the study both before and after the HbA_1c _measurement. Survival analyses and logistic regression were conducted.

**Results:**

Mortality from all causes, cardiovascular and ischaemic heart disease increased with increasing HbA_1c_. Participants with diagnosed diabetes or who had HbA_1c_≥ 7% but no self-reported diabetes had increased mortality risk from all causes and cardiovascular diseases. The respondents in the group HbA_1c _≥7% who had not been diagnosed with diabetes had a significantly higher risk (odds ratio = 4.8 95% CI: 1.1 to 21.6) of developing dementia. Individuals who had self-reported diabetes but a HbA_1c _level <7% had mortality and dementia incidence comparable to individuals without diabetes and HbA_1c _<7%.

**Conclusion:**

The findings support previous reports that bio-markers of glucose metabolism are associated with long term outcomes, such as mortality and dementia.

## Background

Diabetes is already common in the older population and is increasing. In the year 2000, 12% of people aged 65 to 70 years and 15% of people over age 80 have been estimated to have diabetes globally [1]. It is established that diabetes is an independent risk factor for eye, kidney and neurological disease as well as for cardiovascular morbidity and mortality [2]. Two systematic reviews have confirmed its importance as a risk factor for dementia [3,4]. Recent evidence suggests that diabetes is also a risk factor for cognitive dysfunction [3,4] and physical disability [5].

Glucose sticks to the haemoglobin to make a 'glycosylated haemoglobin' molecule, called haemoglobin A1C or HbA_1c _in red blood cells. The more glucose in the blood, the more HbA_1c _will be present in the blood. As red cells live for 8–12 weeks before they are replaced, HbA_1c _level is an indicator of average blood glucose concentration over the past three months rather than blood glucose which fluctuates. The HbA_1c _test is currently one of the clinical methods used to check diabetes control. Clinical practice recommendations suggest a HbA_1c _goal of <7 % [6]. In general, the higher the HbA_1c _value, the higher the risk of having diabetes and of developing complications from diabetes.

Recently the EPIC-Norfolk study, a large epidemiological study, reported a continuous relationship between HbA_1c _and mortality on all cause, cardiovascular, ischaemic heart disease in men and women aged 45 – 79 years old even within the "normal range" of values [7,8]. Shankar and colleagues have also reported that elevated glycosylated haemoglobin levels were associated with all-cause and cardiovascular mortality in type 1 diabetes [9]. There have been inconsistent reports on HbA_1c _levels in relation to cognition. Worrall and colleagues have reported a non significant trend for individuals with extreme lower or higher HbA_1c _levels to have poorer cognitive function as assessed by the Modified Mini-Mental State but not the Delayed Word Recall Test [10]. Two further studies showed no relationship between diabetes and cognition [11,12].

Few studies have investigated the relationship concentrating on the older population. An increasing risk of cognitive decline with age especially among older people is well established, but whether HbA_1c _levels are related to cognitive function and these changes in the older population is not yet clear. Impairment in physical ability is common in old age. Diabetes is a risk factor for impairment of normal physical activity [13], and in one population based study deficits in activities of daily living were reported by 53% of people with diabetes aged 70 years and over [5]. It is uncertain if the relation between HbA_1c _level and conditions associated with diabetes such as vascular disease, cognitive impairment and physical disability shows a threshold effect or whether there is a dose response relationship. This paper investigates the relationship between HbA_1c _levels and mortality, cognitive function and physical disability in the older population in England and Wales.

## Methods

### Study population

**Figure 1:**
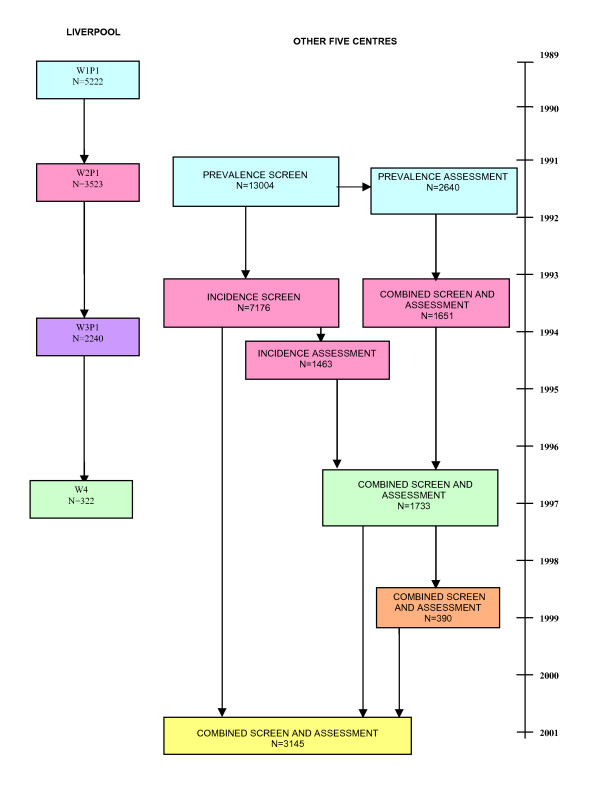
MRC CFAS, flow diagram of study.

The Medical Research Council Cognitive Function and Ageing study (CFAS) is a multi-centre, longitudinal study of people aged 65 years and over (over 18,000 individuals at baseline). It is predominantly a two-phase population based interview study, with annual or biennial follow-ups. There are six CFAS centres, two in rural areas (Cambridgeshire and Gwynedd, Wales) and four in urban areas (Nottingham, Newcastle, Oxford and Liverpool). Details of the CFAS study design have been published previously [14,15] (see Figure 1 for a simplified flow diagram).

### Interviews

Blood was collected at the third wave of interview in 1996 – 1998, between four to six years after the start of the study in all centres except Oxford. All those who had previously received an assessment interview including the study diagnosis were invited to participate in this wave of the study. Sampling at prevalence assessment was weighted towards cognitive impairment. Blood samples were requested from all respondents, and samples were collected from 69% (1,150 out of 1654). An EDTA-anticoagulated blood sample was sent by post to Cambridge, and assayed at the Department of Clinical Biochemistry, University of Cambridge. The HbA1c assays were carried out using high-performance liquid chromatography (HPLC) on a Bio-Rad Diamat Automated Glycosylated Haemoglobin Analyser (Hemel Hempstead, United Kingdom) that was DCCT-aligned. The HbA_1c _readings were fed back to the GPs. 11 samples were undated, and were excluded. The HbA_1c _measurements from 1,139 respondents were used in the analysis. A cut-off point of 7% has been used during this investigation for diagnosis of diabetes, as it has been reported to provide produced the maximum sum of sensitivity and specificity [16] and has been used in similar studies such as EPIC-Norfolk study [8]. The respondents were divided into five categories: those with self reported diabetes, those with a HbA_1c _level ≥7% but who did not report they had been previously diagnosed with diabetes, as we believe that some of the people who were diagnosed with diabetes have been treated and monitored, therefore there will be some different impacts on cognitive function and physical function between these two groups. Three further groups defined by classifying the remaining respondents as low, medium and high tertiles of HbA_1c_, the ranges for the three groups are 3.7%–5.2%, 5.3%–5.7% and 5.8%–6.9%,

Two years after the blood collection interview, a subgroup of respondents was interviewed again, and after another two years, all surviving respondents (excluding Liverpool) were interviewed (year 10 interview). 524 out of the 1139 respondents with a HbA1c measurement had at least one follow up interview. The median follow-up time from blood collection was 5 years.

### Exposure/risk measurement

Information on residence, marital status, main occupation, social and service contacts, physical health and well-being was collected at baseline interview. Change in any of these variables was obtained from follow-up interviews. Data on health conditions including detailed cognitive function and physical function are available both before and after the HbA_1c _measurement in those individual with follow up interviews.

Risk factors for cognitive impairment measured by self report or informant at baseline interview included history of diabetes, heart attack, stroke and hypertension. The participants with self reported diabetes were defined as those answered "Yes" to the question: "Have you ever had sugar diabetes?" in any interviews. Smoking status was classified into three categories: non-smoker (never smoked), ex-smoker (stopped at least 5 years ago) and current smoker.

### Definition outcomes

All participants were flagged for death notification at the Office of National Statistics (ONS). There had been 619 deaths in the HbA_1c_respondents by the end of 2004 (version 8.0 of data). Cause of death was established from death certificates. Cardiovascular death was defined as International Classification of Disease Revision 9 (ICD9) codes 400–438, ischaemic heart disease death as ICD 9 codes 410–414 and cerebro-vascular disease death as ICD 9 codes 430–438 anywhere on the death certificate.

Cognitive function was measured using the Mini Mental State Examination (MMSE) score [17], which tests a range of cognitive abilities and provides a total score from 0 to 30, and dementia of diagnosis using the computerized algorithm AGECAT organicity level of the Geriatric Mental State Examination (GMS Automated Geriatric Examination for Computer Assisted Taxonomy) [18].

Respondents have been classified as demented, not demented and missing diagnosis using the criteria:

• Demented: AGECAT level O3 and above

• Not demented: AGECAT O0, O1, or O2

• Missing: AGECAT level missing

Individuals unable to complete the full interview were diagnosed using vignette, informant information and interviewer observations.

An incident dementia case was defined as a respondent becoming demented after the interview at which blood was collected.

The activities of daily living (ADL) impairment has been defined using the hierarchy of IADL (instrumental activities of daily living) and/or ADL [19] and based upon the concept of interval of need [20]. Participants' mobility was also rated by the interviewer, as usually ambulant non-housebound, usually ambulant housebound, chairfast permanently, and bedfast permanently. An incident ADL impaired case was defined as a respondent becoming impaired after the interview when blood was collected.

Due to differences in study design and data collected, individuals in Liverpool (n = 168) only contributed to the analysis of mortality.

### Statistical methods

Descriptive analysis was carried out to explore the characteristics of the population, the distribution of variables and the relationship between HbA_1c _and vascular diseases risk factors. Analysis of variance (ANOVA) was used to investigate differences on HbA_1c _levels between categories of demographic variables and risk factors. Linear regression was used to investigate trends in continuous and ordinal variables.

Survival analysis was used to investigate the association between HbA_1c _level and mortality from all causes, cardiovascular disease, cerebral disease, ischaemic heart disease and other causes. Cox proportional hazards models were used to determine the contribution of risk factors to mortality. Logistic regression models have been used to examine of relationships between HbA_1c _level and incidence of dementia, and incidence of physical disability. Proportional hazards assumptions were tested using Kaplan-Meier curves. The analysis uses inverse probability weighting to adjust for the study design using methods described previously [21]. Analysis was performed with STATA version 8.

### Ethical approval

All CFAS interviewing and blood collection have been covered under local and multi-centre ethical approval.

All research has been undertaken independently of the funding bodies.

## Results

### Characteristics

HbA_1c _levels ranged from 3.7% to 13.9% with a skewed distribution with median 5.6%, and mean 5.8%. 91% of values were below 7%, and no differences were found between centres (p = 0.83).

Table 1 shows the characteristics of 1,139 respondents categorised by HbA_1c _level and self reported diabetes. Men without previously diagnosed diabetes were more likely to have a HbA_1c _level ≥7%. Respondents in HbA_1c _level ≥7% category were older and had lower MMSE scores. Those respondents who were in either self reported diabetes or HbA_1c _level ≥7% categories were more likely to have IADL and/or ADL impairment. Respondents with diagnosed diabetes were more likely to report having had a heart attack or stroke.

**Table 1:** Characteristics of study population by HbA1c level and self reported diabetes. Values are number (%) in the category unless stated otherwise

Characteristic		HbA1c (%)				Self reported diabetes	
				
		3.7–5.2	5.3–5.7	5.8–6.9	≥7		Total
	Number (%)	372 (33)	345 (30)	287 (25)	36 (3)	99 (9)	1139
**Age **Median (IQR)		78 (73–84)	78 (74–85)	80 (75–86)	83 (77–89)	80 (75–86)	
**MMSE **Median (IQR)		26 (22–28)	25 (23–28)	25 (21–28)	23 (20–25)	25 (22–28)	
**Sex**	Male	154 (41)	139 (40)	125 (43)	20 (56)	49 (49)	487
**Social Class**	I/II	106 (28)	103 (30)	60 (21)	12 (33)	27 (27)	308
	III	182 (49)	155 (45)	154 (54)	14 (39)	45 (46)	550
	IV/V	73 (20)	77 (22)	64 (22)	10 (28)	23 (23)	247
	Missing	11 (3)	10 (3)	9 (3)	0 (0)	4 (4)	34
**Education**	<10 years	243 (65)	249 (72)	208 (72)	24 (67)	68 (69)	792
**Smoking status**	Current smoker	46 (13)	49 (14)	68 (24)	6 (17)	20 (20)	189
	Ex-smoker	197 (53)	154 (44)	128 (44)	18 (50)	44 (45)	541
	Non smoker	124 (33)	140 (41)	90 (31)	12 (33)	28 (28)	336
	Missing	5 (1)	2 (1)	1 (1)	0 (0)	3 (3)	11
**ADL**	No impairment	206 (55)	177 (51)	138 (48)	11 (31)	34 (34)	566
	IADL impairment	61 (17)	62 (18)	55 (19)	9 (25)	19 (19)	206
	IADL and/or ADL impairment	93 (25)	93 (27)	78 (27)	16 (44)	41 (42)	321
	Missing	12 (3)	13 (4)	16 (6)	0 (0)	5 (5)	46
**History of Heart Attack**		45 (12)	44 (13)	40 (14)	3 (8)	20 (21)	152
**History of Stroke**		36 (10)	42 (12)	36 (13)	4 (11)	21 (21)	139
**History of High Blood Pressure**		126 (34)	142 (41)	116 (40)	18 (50)	42 (42)	444

The mean value of HbA_1c _for smokers and those who reported high blood pressure was systematically higher in the 1,004 respondents whose HbA1c was under 7%. Otherwise there were no systematic or significant differences between the groups with 'normal' HbA_1c _levels.

### Mortality

By the end of 2004, 54% of the 1139 respondents had died. The number of deaths was similar in each the first five follow up years, and increased sharply thereafter. The median follow up time was 3 years from blood collection and interquartile range was 1.4 to 5.0 years for those who had died.

**Figure 2:**
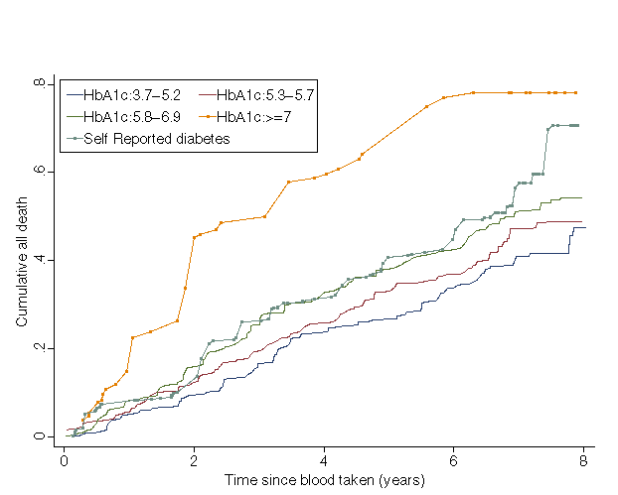
All-cause mortality by five categories of respondents.

Figure 2 displays the cumulative mortality curves from all cause deaths by five categories of respondents: those with self reported diagnosed diabetes, those without diagnosed diabetes but had HbA_1c _≥7% and the three tertiles of the population with HbA1c<7%. Individuals without diagnosed diabetes but who had HbA_1c _≥7% had higher mortality than all others. Table 2 shows age and sex adjusted mortality hazard ratios for these groups. Respondents whose HbA_1c _was greater or equal to 7% but had not previously been diagnosed with diabetes had twice the rate of death from all causes and dying from cardiovascular disease compared with respondents whose HbA_1c _was in the lowest tertile. Respondents who reported diabetes also had nearly double the death rate from cardiovascular disease compared with respondents whose HbA_1c _was in the lowest tertile. The relative risks of ischaemic heart disease and cerebro vascular disease mortality were higher in the respondents who reported diabetes or whose HbA_1c _was greater or equal to 7% but had no self-reported diabetes than the lowest HbA_1c _tertile, although they are not significant at 5% level. There was no significant difference between the three groups in which respondents' HbA_1c _was less than 7%, although there may be a slight trend with increasing tertiles. Adjusting for smoking status did not change the patterns in the mortality hazard ratios.

**Table 2:** Age and sex adjusted hazard ratios

Cause of death	HbA1c (%)				Self reported
		
	T1 (3.7–5.2)	T2 (5.3–5.7)	T3 (5.8–6.9)	≥7	Diabetes
	
	(n = 372)	(n = 345)	(n = 287)	(n = 36)	(n = 99)
All causes (n = 619)	176	177	170	27	69
Hazard ratio (95% CI)	1.0	1.1 (0.8,1.5)	1.3 (0.9,1.8)	2.0 (1.1,3.9)	1.4 (0.8 2.3)
Cardiovascular disease (n = 316)	86	86	88	15	41
Hazard ratio (95% CI)	1.0	1.1 (0.7,1.8)	1.4 (0.9,2.2)	2.1 (1.0,4.4)	1.9 (1.0,3.5)
Ischaemic heart disease (n = 165)	44	45	47	6	23
Hazard ratio (95% CI)	1.0	1.2 (0.7,2.1)	1.4 (0.8 2.5)	1.6 (0.5,5.5)	1.8 (0.8,4.0)
Cerebro vascular disease (n = 120)	34	37	26	6	17
Hazard ratio (95% CI)	1.0	1.1 (0.5,2.2)	1.1 (0.5,2.3)	2.5 (0.8,8.0)	1.7 (0.7,4.2)
Non-cardiovascular disease (n = 303)	90	91	82	12	28
Hazard ratio (95% CI)	1.0	1.1 (0.7,1.7)	1.1 (0.7,1.8)	1.8 (0.7,5.1)	1.0 (0.5,2.0)

Table 3 shows the relations between HbA_1c _level and/or diabetes status and mortality from all cause, cardiovascular, ischaemic, cerebro vascular and non-cardiovascular disease modeled using Cox multivariable regression models adjusted for age and sex only and additionally for history of heart attack, stroke, hypertension and smoking status respectively. There is a continuous relationship between HbA_1c _and mortality on all cause, cardiovascular and ischaemic heart disease after taking of account of age, sex and some known risk factors even including diabetes status. An increase of 1% in HbA_1c _was associated with about 10% (95% CI: 1% to 24%) increase in all cause mortality and 20% increase in cardiovascular (95% CI: 7% to 37%) or ischaemic disease (95% CI: 4% to 44%) mortality. However there was no significant association between self reported diabetes status and mortality. The risk associated with HbA1c is independent of self-reported diabetes. After excluding the respondents with self-reported diabetes or with a HbA_1c _level ≥ 7%, the relative risks of all cause, cardiovascular and ischaemic heart disease were similar, but were no longer significant.

**Table 3:** Cox multivariate regression for all cause, cardiovascular, ischaemic heart disease, cerebro disease and non-cardiovascular mortality. Effect of HbA1c level and diabetes status were modelled separately (models 1 and 2) and together (model 3)

Cause of death		Relative risk adjusted for age & sex (95% CI)	P value	Relative risk adjusted for age, sex and other risk factors* (95% CI)	P value
All causes (n = 619)					
Model 1	HbA_1c _(per 1% increase)	1.1 (1.0,1.3)	0.03	1.1 (1.0, 1.2)	0.03
Model 2	Diabetes history (yes v no)	1.2 (0.8, 1.8)	0.38	1.2 (0.8, 1.9)	0.44
Model 3	HbA_1c _(per 1% increase)	1.2 (1.0, 1.3)	0.04	1.2 (1.0, 1.4)	0.08
	Diabetes history (yes v no)	0.9 (0.6, 1.4)	0.67	0.8 (0.4, 1.6)	0.61
Cardiovascular disease (n = 316)					
Model 1	HbA_1c _(per 1% increase)	1.2 (1.1, 1.4)	0.001	1.2 (1.1 1.4)	0.002
Model 2	Diabetes history (yes v no)	1.6 (1.0, 2.6)	0.056	1.5 (0.1, 2.6)	0.10
Model 3	HbA_1c _(per 1% increase)	1.2 (1.1, 1.4)	0.01	1.2 (1.1, 1.5)	0.01
	Diabetes history (yes v no)	1.1 (0.6, 1.8)	0.79	0.9 (0.5, 1.8)	0.81
Ischaemic heart disease (n = 165)					
Model 1	HbA_1c _(per 1% increase)	1.2 (1.1, 1.5)	0.01	1.2 (1.0, 1.4)	0.01
Model 2	Diabetes history (yes v no)	1.7 (1.0, 3.1)	0.08	1.5 (0.8, 3.0)	0.24
Model 3	HbA_1c _(per 1% increase)	1.2 (1.0, 1.5)	0.06	1.3 (1.0, 1.6)	0.03
	Diabetes history (yes v no)	1.2 (0.6, 2.1)	0.63	0.8 (0.4, 1.9)	0.68
Cerebro disease (n = 120)					
Model 1	HbA_1c _(per 1% increase)	1.2 (1.0, 1.5)	0.08	1.1 (0.9, 1.5)	0.29
Model 2	Diabetes history (yes v no)	1.6 (0.8, 3.3)	0.17	1.4 (0.7, 2.7)	0.40
Model 3	HbA_1c _(per 1% increase)	1.2 (0.9, 1.5)	0.29	1.1 (0.8, 1.6)	0.42
	Diabetes history (yes v no)	1.2 (0.6, 2.8)	0.61	1.0 (0.4, 2.4)	0.97
Non-cardiovascular disease (n = 303)					
Model 1	HbA_1c _(per 1% increase)	1.0 (0.9, 1.2)	0.87	1.0 (0.8, 1.2)	0.97
Model 2	Diabetes history (yes v no)	0.8 (0.5, 1.6)	0.58	0.8 (0.4, 1.7)	0.65
Model 3	HbA_1c _(per 1% increase)	1.1 (0.9, 1.4)	0.57	1.1 (0.8, 1.4)	0.68
	Diabetes history (yes v no)	0.8 (0.4, 1.6)	0.46	0.7 (0.2, 2.2)	0.58

* Risk factors are history of heart attack, stroke, high blood pressure and smoke status

There were 99 people with self reported diagnosed diabetes and of these one third had HbA_1c _concentration levels below 7%. To examine whether this group of respondents differed with respect to mortality from respondents who reported diabetes but had HbA_1c _concentration levels above 7%, we separated the respondents into four groups:

• HbA_1c _< 7% without self reported diagnosis of diabetes

• HbA_1c _≥ 7% and no self reported diabetes

• reported diagnosed diabetes and HbA_1c _< 7%

• reported diagnosed diabetes and HbA_1c _≥ 7%

Table 4 shows that respondents who had HbA_1c _≥ 7% in self reported diabetes group had significant higher hazard rate of dying from cardiovascular disease and ischaemic heart disease compared with the group of respondents who had HbA_1c _< 7% and without self reported diagnosis of diabetes even after adjusting for age and sex. Individuals who reported diabetes but were well controlled according to HbA_1c _level had similar rates of death compared with individuals who do not have diabetes, although the confidence intervals are wide.

**Table 4:** Age and sex adjusted hazards ratios

	Not self reported diabetes		Self reported diabetes	
	
	HbA1c (%)<7	HbA1c (%)≥7	HbA1c (%)<7	HbA1c (%)≥7
Cause of death	(n = 1004)	(n = 36)	(n = 36)	(n = 63)
All causes (n = 619)	523	27	25	44
Hazard ratio (95% CI)	1.0	1.8 (0.9,3.3)	1.2 (0.4,3.2)	1.3 (0.8,2.1)
Cardiovascular disease (n = 316)	260	15	10	31
Hazard ratio (95% CI)	1.0	1.9 (0.9,3.7)	0.5 (0.2,1.6)	2.1(1.2,3.7)
Ischaemic heart disease (n = 165)	136	6	6	17
Hazard ratio (95% CI)	1.0	1.4 (0.4,4.6)	0.5 (0.1,1.7)	2.1 (1.0,4.3)
Cerebro disease (n = 120)	97	6	3	14
Hazard ratio (95% CI)	1.0	2.4 (0.8,7.1)	0.6 (0.1,2.5)	2.0 (0.8,4.6)
Non-cardiovascular disease (n = 303)	263	12	15	13
Hazard ratio (95% CI)	1.0	1.7 (0.7,4.5)	1.8 (0.6,5.4)	0.6 (0.2,1.4)

### HbA_1c _and dementia

In this sample, dementia increased with age and was more common in women (9.5%) than men (2.6%). There was no cross sectional association between HbA_1c _level and prevalent dementia, adjusted for sex and age at baseline.

There were 67 incident cases of dementia identified in 453 individuals during an average 5 years follow up since HbA1c measurement. Dementia in this sample increased with age and more women developed dementia (14.6%) than men (4.9%). Table 5 shows the percentage of respondents developing dementia over the 5 years, in HbA_1c _≥ 7% category this percentage was considerably higher than other groups. The percentages in the three other HbA_1c _groups were similar. The percentage developing dementia in the self reported diabetes group was low. The crude death rate by 5 years was 70% among people with self reported diabetes compared to 52% in participants with HbA_1c _level < 7%.

**Table 5:** Percentages of individuals developing dementia over 5 years follow-up within each HbA1c group

	HbA1c (%)				Self reported
		
	T1 (3.7–5.2)	T1 (5.3–5.7)	T1 (5.8–6.9)	≥7^a^	Diabetes
Total	372	345	287	36	99
Number of Cases^b^	62	63	43	14	10
Cumulative Incidence^b^	10.7	10.9	11.8	49.1	7.5
95% CI^b^	(5.3,20.4)	(5.9,19.2)	(6.1,21.7)	(14.7,84.4)	(1.9,16.8)

^a ^Not self-reported diabetes

^b ^Number of cases, incidence and CI are back-weighted to the population sample

A logistic regression model found that respondents in the group HbA_1c _≥ 7% who had not been diagnosed with diabetes had significant higher risk (odds ratio = 4.8, 95% CI:1.1 to 21.6) of developing dementia than the respondents with HbA_1c _< 7% even after adjusting for age and sex.

### HbA_1c _and disability

Table 1 shows that respondents who were in both self reported diabetes category and no self reported diabetes but with HbA_1c _level ≥7% category were more likely to have IADL and/or ADL impairment at the time blood was collected, even after adjusting for age and sex. But there was no relationship between IADL and/or ADL impairment and levels of HbA_1c_under 7%.

114 respondents with no IADL or ADL impairment at the time of blood collection had developed one in the five follow up years. Overall there was no relationship between the incidence of IADL and/or ADL impairment and HbA_1c _level or diabetes status. Women with HbA_1c _level ≥7% had 60 % higher risk than women with HbA_1c _level in the lower tertiles after adjusting for age, but confidence intervals are wide (95% CI: 0.3, 9.5).

## Discussion

The aim of this study is to investigate the relationship between HbA_1c_levels and mortality, cognitive function and physical disability in the older population in England and Wales. It was found that there was a continuous relationship between HbA1c level and mortality from all cause, cardiovascular and ischaemic heart disease. Respondents who did not report diabetes but had HbA_1c _≥7 % had the highest mortality rate from all causes and cardiovascular disease independent of age and sex compared with respondents in the lowest HbA1c tertile. Respondents with a known history of diabetes were at an increased risk of dying from cardiovascular disease independent of age and sex. However respondents with known history of diabetes, but with HbA_1c _under 7% had similar rates of the mortality to respondents without diabetes whose HbA_1c _level were also under 7%. Individuals with poorly controlled known diabetes had the highest mortality risk.

In the main analysis of mortality, we adjusted only age and sex. In order to see if confounding factors would influence the results, we also performed sensitivity analysis adjusted for all the available risk factors in our study such as history of heart attack, stroke, high blood pressure and smoke status, and there were no significant changes. We would have liked to include some possible confounding factors such as BMI, but unfortunately the information was not collected in our study.

In contrast to established literature this analysis did not show that individuals with diagnosed diabetes were at higher risk of developing dementia [5]. There was no evidence to suggest that HbA_1c _level was related to the current dementia. However, there was a significant increase in the risk of developing dementia for individuals with HbA_1c _≥7 % but who did not report they had been previously diagnosed with diabetes. It may be that some of the people who would have developed dementia had they survived were differentially lost to follow up because of premature mortality due to diabetes. Since overall prevalence of dementia at death was 30% and there was a strong increasing trend for dementia with age from 6% for those aged 65–69 years old at time of death to 58% for those aged 95 years old and above at time of death [22], there might be more incident cases missed from this high mortality group. In addition, the low incidence rate in this group might well be by chance.

However, considering that the mortality in HbA_1c _≥ 7% but no self-reported diabetes category was high, these biases would imply that the incidence rate of dementia would be even higher than observed. Figure 2 shows that a higher early mortality from all-cause, and particularly from non-cardiovascular causes was seen in those with HbA_1c _≥ 7%, compared to the other groups (including self-reported diabetes). These findings support the observation that at diagnosis of diabetes half already have clinical evidence of diabetic tissue damage and that cardiovascular risk factors are common [23,24]

The relative risks in our study were not as substantial as those found elsewhere [7,8]. This may be due to the differences in the populations studied, and attrition of effects with age. In the EPIC-Norfolk study, the respondents are much younger (45–79 years) compared with CFAS where the respondents were aged 69–103 years. There were continuous relationships between increasing HbA_1c _level and the increasing risks of death from all cause, cardiovascular and ischaemic heart disease after taking into account of age, sex and some known risk factors even including diabetes status in the models, though no relationship was found to subsequent non cardiovascular disease and cerebrovascular disease deaths.

Individuals who had a HbA_1c _≥ 7% were informed via their GP of this fact, though we did not specifically follow up on treatment or confirmation of the diagnosis within these individuals. Information was requested about diabetes history at the next routine interview (four years later). Some individuals will have been treated, therefore it could be that we have under estimated the morbidity/mortality of undiagnosed diabetes.

Increasing HbA_1c _level was not associated with risk of developing IADL and/or ADL impairment in the whole sample population, but it was associated with an increasing risk of developing IADL and/or ADL impairment in women. We also have found the evidence to support previous literature that community-living older subjects with diabetes have high rates of deficits in physical function [5], however, our results showed that the incidence rates are similar in each category, this possibly due to a small number of diabetic patients in our sample and older age groups with increasing impairments in the whole population.

Missing data at the longitudinal follow-ups are a potential problem for all longitudinal studies. Excluding deaths, 25% of individuals with a HbA_1c _measurement did not have a follow-up interview. This could be a problem for estimation of incidence of dementia as this is associated with dropout [25]. If this relationship did exist, it would accentuate the differences between those with and without cognitive decline as those with cognitive decline would be missed. However this will only affect the estimates if dropout is independently related to HbA_1c _levels or diabetes. There is no evidence to suggest this [25].

Various HbA_1c _thresholds have been proposed for indicating blood sugar control. We have used 7% as this was the recommended level at the time of the study. This will not be consistent with other studies [26]. The classification of diabetes will therefore be different to some reported findings [27]. In addition, respondents were divided into three groups using tertiles of HbA1c for those with HbA1c under 7 %, which caused different cut points from some studies such as the one used in EPIC-Norfolk. Using EPIC-Norfolk [7,8] cut points in the survival analysis may make the results more easily comparable with this study, however these cut points were based on measurements of participants with a very different age range (45–79 years).

Individuals who had self-reported diabetes but whose HbA_1c _was under 7% had mortality and dementia incidence comparable to individuals without diabetes and HbA_1c _<7%. While these results suggest that treating diabetes reduces risk of mortality and dementia, other explanations for our findings need to be considered.

The association between abnormal glucose regulation and cardiovascular disease is well known. However, this may not be a direct causal association. It is hypothesized that the constellation of cardiovascular risk factors that constitute the metabolic syndrome (elevated glucose, hypertension, dyslipidaemia and central obesity) share a common causal factor. Insulin resistance is a typical feature of individuals with the metabolic syndrome [28]. HbA_1c _could therefore be a risk marker for other cardiovascular risk factors.

Alternatively, there are mechanisms that may directly link HbA_1c _with dementia and cardiovascular disease. Advanced glycated end products are formed when proteins are exposed to glucose in a process analogous to formation of HbA_1c _where glucose binds to haemoglobin_. _They have been implicated in diabetic vascular disease [29] and may be related to the development of Alzheimer's Disease [30].

## Conclusion

The findings from this study provide further evidence for HbA_1c _as an indicator of future health. To control HbA_1c _level effectively under 7% may reduce the risks of dying from cardiovascular disease and ischaemic heart disease and contribute to maintenance of cognitive function in the older population.

## Competing interests

The author(s) declare that they have no competing interests.

## Authors' contributions

LG carried out the statistical analysis and drafted the manuscript. FM participated in the study design, analysis and helped to the draft the manuscript. LS participated in the drafting the manuscript and revising it critically for important clinical content. CB conceived the study, and participated in its design and coordination and helped to draft the manuscript. All authors read and approved the final manuscript.

## Pre-publication history

The pre-publication history for this paper can be accessed here:



## References

[B1] Wild S, Roglic G, Green A, Sicree R, King H (2004). Global prevalence of diabetes: estimates for the year 2000 and projections for 2030. Diabetes Care.

[B2] Weatherall DJ, Ledingham GG, warrell DA (1996). Oxford textbook of medicine.

[B3] Cukierman T, Gerstein HC, Williamson JD (2005). Cognitive decline and dementia in diabetes--systematic overview of prospective observational studies. Diabetologia.

[B4] Biessels GJ, Staekenborg S, Brunner E, Brayne C, Scheltens P (2006). Risk of dementia in diabetes mellitus: a systematic review. Lancet Neurol.

[B5] Bruce DG, Casey GP, Grange V, Clarnette RC, Almeida OP, Foster JK, Ives FJ, Davis TM (2003). Cognitive impairment, physical disability and depressive symptoms in older diabetic patients: the Fremantle Cognition in Diabetes Study. Diabetes Res Clin Pract.

[B6] (2006). Standards of medical care in diabetes--2006. Diabetes Care.

[B7] Khaw KT, Wareham N, Luben R, Bingham S, Oakes S, Welch A, Day N (2001). Glycated haemoglobin, diabetes, and mortality in men in Norfolk cohort of european prospective investigation of cancer and nutrition (EPIC-Norfolk). BMJ.

[B8] Khaw KT, Wareham N, Bingham S, Luben R, Welch A, Day N (2004). Association of hemoglobin A1c with cardiovascular disease and mortality in adults: the European prospective investigation into cancer in Norfolk. Ann Intern Med.

[B9] Shankar A, Klein R, Klein BE, Moss SE (2007). Association between glycosylated hemoglobin level and cardiovascular and all-cause mortality in type 1 diabetes. Am J Epidemiol.

[B10] Worrall GJ, Chaulk PC, Moulton N (1996). Cognitive function and glycosylated hemoglobin in older patients with type II diabetes. J Diabetes Complications.

[B11] Lowe LP, Tranel D, Wallace RB, Welty TK (1994). Type II diabetes and cognitive function. A population-based study of Native Americans. Diabetes Care.

[B12] Robertson-Tchabo EA, Arenberg D, Tobin JD, Plotz JB (1986). A longitudinal study of cognitive performance in noninsulin dependent (type II) diabetic men. Exp Gerontol.

[B13] Gregg EW, Beckles GL, Williamson DF, Leveille SG, Langlois JA, Engelgau MM, Narayan KM (2000). Diabetes and physical disability among older U.S. adults. Diabetes Care.

[B14] (1998). Cognitive function and dementia in six areas of England and Wales: the distribution of MMSE and prevalence of GMS organicity level in the MRC CFA Study. The Medical Research Council Cognitive Function and Ageing Study (MRC CFAS). Psychol Med.

[B15] Yip AG, Brayne C, Easton D, Rubinsztein DC (2002). Apolipoprotein E4 is only a weak predictor of dementia and cognitive decline in the general population. J Med Genet.

[B16] McCance DR, Hanson RL, Charles MA, Jacobsson LT, Pettitt DJ, Bennett PH, Knowler WC (1994). Comparison of tests for glycated haemoglobin and fasting and two hour plasma glucose concentrations as diagnostic methods for diabetes. BMJ.

[B17] Folstein MF, Folstein SE, McHugh PR (1975). "Mini-mental state". A practical method for grading the cognitive state of patients for the clinician. J Psychiatr Res.

[B18] Copeland JR, Dewey ME, Griffiths-Jones HM (1986). A computerized psychiatric diagnostic system and case nomenclature for elderly subjects: GMS and AGECAT. Psychol Med.

[B19] Spector WD, Fleishman JA (1998). Combining activities of daily living with instrumental activities of daily living to measure functional disability. J Gerontol B Psychol Sci Soc Sci.

[B20] Isaacs B, Neville Y (1976). The needs of old people. The 'interval' as a method of measurement.. Br J Prev Soc Med.

[B21] Yip AG, Brayne C, Matthews FE (2006). Risk factors for incident dementia in England and Wales: The Medical Research Council Cognitive Function and Ageing Study. A population-based nested case-control study. Age Ageing.

[B22] Brayne C, Gao L, Dewey M, Matthews FE, Medical Research Council Cognitive Function and Ageing Study Investigators. (2006). Dementia before death in ageing societies--the promise of prevention and the reality.. PLoS Med.

[B23] (1990). UK Prospective Diabetes Study 6. Complications in newly diagnosed type 2 diabetic patients and their association with different clinical and biochemical risk factors. Diabetes Res.

[B24] Haffner SM, Stern MP, Hazuda HP, Mitchell BD, Patterson JK (1990). Cardiovascular risk factors in confirmed prediabetic individuals. Does the clock for coronary heart disease start ticking before the onset of clinical diabetes?. JAMA.

[B25] Matthews FE, Chatfield M, Freeman C, McCracken C, Brayne C (2004). Attrition and bias in the MRC cognitive function and ageing study: an epidemiological investigation. BMC Public Health.

[B26] Peters AL, Davidson MB, Schriger DL, Hasselblad V (1996). A clinical approach for the diagnosis of diabetes mellitus: an analysis using glycosylated hemoglobin levels. Meta-analysis Research Group on the Diagnosis of Diabetes Using Glycated Hemoglobin Levels. JAMA.

[B27] Engelgau MM, Thompson TJ, Herman WH, Boyle JP, Aubert RE, Kenny SJ, Badran A, Sous ES, Ali MA (1997). Comparison of fasting and 2-hour glucose and HbA1c levels for diagnosing diabetes. Diagnostic criteria and performance revisited. Diabetes Care.

[B28] Lebovitz HE (2006). Insulin resistance--a common link between type 2 diabetes and cardiovascular disease. Diabetes Obes Metab.

[B29] Goldin A, Beckman JA, Schmidt AM, Creager MA (2006). Advanced glycation end products: sparking the development of diabetic vascular injury. Circulation.

[B30] Schmitt HP (2006). epsilon-Glycation, APP and Abeta in ageing and Alzheimer disease: a hypothesis. Med Hypotheses.

